# Size-Dependent Role of Surfaces in the Deformation of Platinum Nanoparticles

**DOI:** 10.1021/acsnano.2c11457

**Published:** 2023-04-26

**Authors:** Soodabeh Azadehranjbar, Ruikang Ding, Ingrid M. Padilla Espinosa, Ashlie Martini, Tevis D. B. Jacobs

**Affiliations:** † Department of Mechanical Engineering and Materials Science, University of Pittsburgh, Pittsburgh, Pennsylvania 15261, United States; ‡ Department of Mechanical Engineering, University of California, Merced, Merced, California 95340, United States

**Keywords:** platinum nanoparticles, in situ TEM, displacive deformation, diffusive deformation, nanomechanical testing

## Abstract

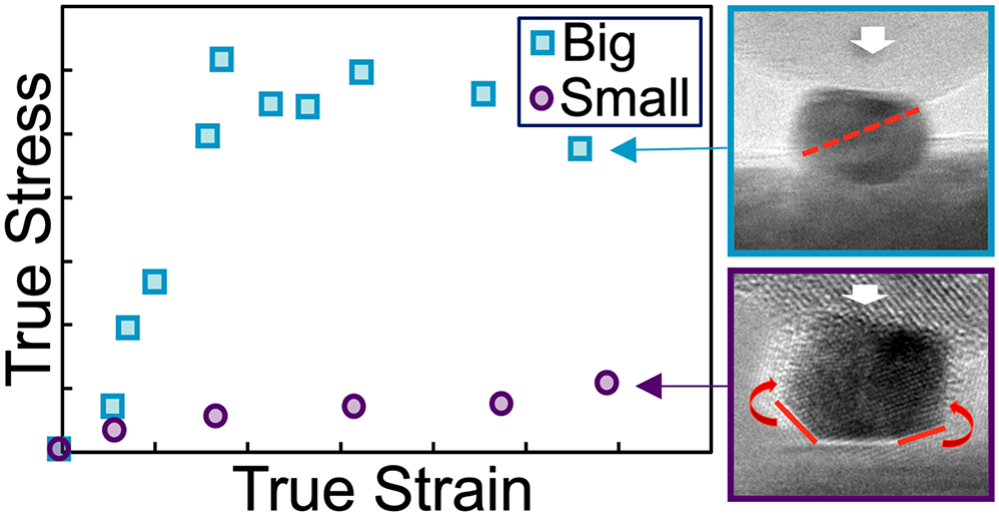

The mechanical behavior of nanostructures is known to transition from a Hall-Petch-like “smaller-is-stronger” trend, explained by dislocation starvation, to an inverse Hall-Petch “smaller-is-weaker” trend, typically attributed to the effect of surface diffusion. Yet recent work on platinum nanowires demonstrated the persistence of the smaller-is-stronger behavior down to few-nanometer diameters. Here, we used in situ nanomechanical testing inside of a transmission electron microscope (TEM) to study the strength and deformation mechanisms of platinum nanoparticles, revealing the prominent and size-dependent role of surfaces. For larger particles with diameters from 41 nm down to approximately 9 nm, deformation was predominantly displacive yet still showed the smaller-is-weaker trend, suggesting a key role of surface curvature on dislocation nucleation. For particles below 9 nm, the weakening saturated to a constant value and particles deformed homogeneously, with shape recovery after load removal. Our high-resolution TEM videos revealed the role of surface atom migration in shape change during and after loading. During compression, the deformation was accommodated by atomic motion from lower-energy facets to higher-energy facets, which may indicate that it was governed by a confined-geometry equilibration; when the compression was removed, atom migration was reversed, and the original stress-free equilibrium shape was recovered.

## Introduction

Metal nanoparticles are used in technology applications, such as sensing,^1,2^ catalysis,^3,4^ optoelectronics,^5^ and microelectromechanical systems.^6^ Their reliability in these applications depends on their stability and mechanical behavior and thus requires a clear understanding of their deformation under load. It is well established that decreasing the size of a structure increases its strength by reducing or eliminating defects and defect sources, giving rise to the “smaller-is-stronger” trend.^7−10^ Below the size scale of a few hundred nanometers, surface nucleation of dislocations^11^ becomes the dominant deformation process in pristine nanostructures^12−14^ as well as twinned and multimetallic structures,^15^ resulting in a size-independent high-strength plateau.^13,16−18^ This surface nucleation has been extensively studied in nanowires and can be described as a stress-dependent thermally activated process,^12^ where the activation barrier is highly sensitive to the surface state, with surface steps and terraces lowering the yield strength.^19−21^

It was later demonstrated that plastic deformation can occur in the absence of defect-based mechanisms, with shape change instead occurring through long-range atom migration.^22,23^ At sufficiently high homologous temperatures^24,25^ or ultrasmall sizes,^25^ surface diffusion is proposed to provide an alternative mechanism for shape rearrangement under stress. This mechanism has been described using the framework of Coble creep^24,25^ with the stress proportional to strain rate and inversely proportional to the cube of nanoparticle size.^24,26^ This so-called *diffusive plasticity* manifests as a homogeneous, liquid-like response to loading, even though the nanoparticle or nanowire remains crystalline.^26,27^ Furthermore, structures deformed in this way can spontaneously recover their original shape after compression as observed in the blunting of fractured Cu nanowires^24^ and liquid-like deformation of 10 nm Ag particles.^26^ The behavior is often called pseudoelasticity or shape recovery. However, unlike investigations showing shape recovery by the reverse motion of defects (e.g., due to interactions with twin boundaries^28^ or due to reversible twinning and detwinning^29^), surface-diffusion-induced shape recovery is mainly attributed to the curvature-driven surface diffusion of atoms.^24^

Very recently, an in situ TEM investigation measured the failure stress of Ag and Pt nanowires and observed their deformation mechanisms.^30^ For Ag, that work showed that the traditional Hall-Petch-like trend continued until reaching peak strength at nanowire diameters of approximately 15 nm, below which an inverse Hall-Petch trend was observed. By contrast, the results for Pt demonstrated that the Hall-Petch-like trend persisted at the very smallest sizes. In interpreting these results, the study attributed the weakening of Ag not to the long-range atom migration assumed by the Coble-creep-like model but rather to surface-diffusion-induced acceleration of the surface-nucleation of dislocations. The authors referred to this as *diffusion-assisted dislocation nucleation*. Smaller-diameter wires exhibited faster diffusion which increased the chance of nucleation, and thus the strength decreased. However, this smaller-is-weaker trend never appeared in Pt, which the authors attributed to slower diffusion due to its high melting temperature. Large-scale deformation via surface diffusion was not observed in any of the wires, and all deformation was attributed to displacive mechanisms that were assisted by diffusion. This agrees with other findings suggesting defect-based plasticity even at the smallest sizes in gold.^31^

Given the discrepancies between the expected Coble-creep-like deformation and the observed diffusion-assisted displacive deformation in platinum nanowires, the purpose of this investigation was to interrogate the competing effects of surfaces in nanoparticle deformation: accommodating shape change through atomic migration and facilitating surface nucleation of dislocations. To accomplish this, in situ compression tests were performed on Pt nanoparticles with diameters ranging from 41 to 6 nm. The tests were performed in a TEM using an atomic-force-microscopy probe as the indenter (see Methods) enabling real-time measurement of the load, deformation, shape, and structure of the particles during testing.

## Results and Discussion

### Size-Dependent Mechanical Behavior of Pt Nanoparticles

**Figure 1:**
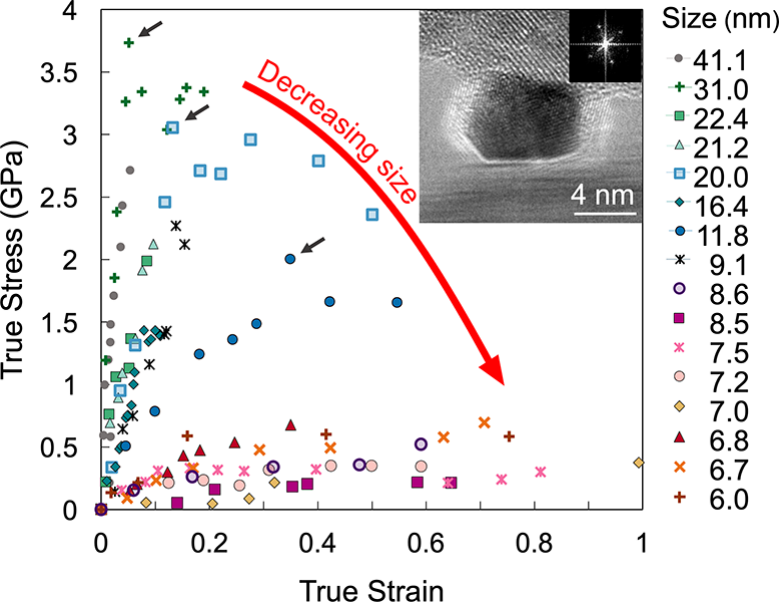
In situ compression tests of particles with diameters ranging from 6 to 41 nm demonstrated clear size-dependent behavior. Particles with diameters larger than 9 nm exhibited approximately linear behavior up to a peak (black arrows), followed by a plateau. Particles with diameters less than 9 nm showed gradual deformation at lower stresses to far larger strains. The inset shows a nanoparticle being compressed in the TEM.

The mechanical behavior of the nanoparticles is shown using true-stress–true-strain curves in Figure 1, where tests showed a range of peak stresses from a maximum of 3.7 GPa to a minimum of 220 MPa. As described in the Methods, the stress was computed as the instantaneous force over the instantaneous contact area between the particle and the indenter, while the strain was computed as the logarithm of the ratio of instantaneous height to initial height. Two distinct shapes of the stress–strain curve were observed for nanoparticles of different sizes. For particles larger than 9 nm, there was linear-elastic behavior to more than 1 GPa with a clear peak (black arrows in Figure 1). Some of these tests were stopped in the linear regime (e.g., the 41-nm particle); but, for those that were not stopped (e.g., the 31-nm particle), the peak stress was followed by a plateau during which yielding continued. By contrast, for particles smaller than 9 nm, the peak stress was significantly lower (<700 MPa) as compared to large particles and substantial strain was exhibited, up to a maximum of 1.0. No clear linear-elastic behavior was observed, nor was there a kink or transition between lower-strain and higher-strain trends in the data.

**Figure 2:**
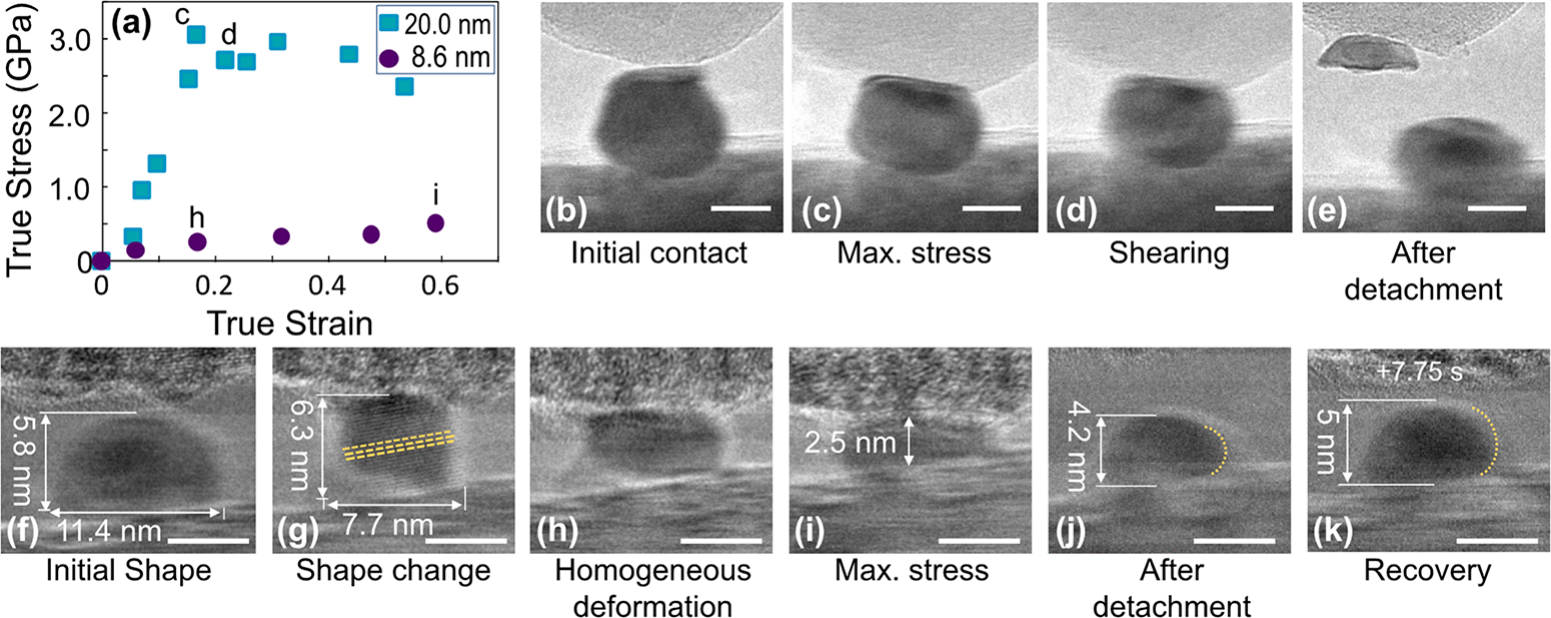
Stress–strain (a) and deformation (b–k) are compared for two representative particles. A 20-nm particle exhibited high strength and localized deformation, with video frames shown at initial contact (b), max stress (c), during deformation (d), and after testing (e). An 8.6-nm Pt nanoparticle failed at low strength and is shown in its initial state (f) and during gradual deformation (g–i) with lattice fringes confirming the crystallinity of the particle. The particle is also shown immediately after testing (j) and 7.75 s after load removal (k) as shape recovery continues. Scale bars are 10 nm in (b,c) and 5 nm in (f–k).

To understand the distinct behavior of these two stress–strain trends, we examined a representative particle from each category in detail (Figure 2). Figure 2a shows the true-stress–true-strain curves for the 20-nm particle and the 8.6-nm particle. The 20-nm particle exhibited high strength and a clear transition between elastic and post-elastic straining, as shown in Figure 2b–e and in Supporting Information Video S1. The particle was linearly compressed up to a peak stress of 3.1 GPa (point (c) in Figure 2a). Then, after the peak, there was a yielding plateau starting at point d, which, in the video and still frames (Figure 2d), corresponded to shearing and total failure of the particle (Figure 2e). This kink in the stress–strain curve and localized shearing (inhomogeneous deformation) is characteristic of displacive deformation, in which the deformation is carried primarily by dislocations. Prior investigations by others on nanowires^12,32−34^ and earlier work on platinum^35^ and palladium^36^ nanoparticles have shown that defect plasticity in face-centered cubic (FCC) nanostructures proceeds via Shockley partial dislocations that leave behind stacking faults. However, the present testing was optimized for load measurement and the specific defects were not identified. The yield strength of this particle, 3.1 GPa, was much higher than that of bulk Pt (of order 100 MPa), but significantly lower than Pt’s theoretical strength of 9.5 GPa^17^ and previous measurements of nanowires in the range of 4.5–9 GPa.^27^ In the end, this particle failed inhomogeneously and separated with part of the particle attached to the indenter. While the separation plane appears to be parallel to the indenter surface, our prior work on similar particles indicates failure along the expected Schmid-factor plane;^35^ therefore, we attribute the present shape to post-test flattening when the top portion of the particle slid off the lower portion and hit the substrate. The majority of the larger particles failed along an inclined plane, similar to those shown in Supporting Information Section S2.

The 8.6-nm nanoparticle exhibited very different behavior, showing no well-defined yield strength and exhibiting dramatic plastic flow at relatively low stress levels (<0.5 GPa). The morphological evolution of this particle is shown in Figure 2f–i (snapshots from Supporting Information Video S2). Immediately upon contact, the particle exhibited a shape change (Figure 2f,g), showing an immediate increase in height and decrease in width. This initial shape change is attributed to adhesion of the particle to the indenter surface. It is likely that an instantaneous vibration caused the particle and substrate to come into contact at a moment when there was still a finite gap between them; because of adhesion, this briefly put the particle in tension and caused it to elongate. This is consistent with similar adhesion and lengthening of nanoparticles in prior work.^26^ This “liquid-like” deformation continued homogeneously throughout the compression test, yet lattice fringes corresponding to {111} planes (yellow lines in Figure 2g) confirm the crystallinity of the particle during the substantial shape change up to 60% strain. During unloading, the nanoparticle recovered its original sphere-like shape and recovered from a minimum height of 2.5 nm to a height of 4.2 nm upon load removal. This height recovery continued over time after separation (Figure 2k). Reversible shape recovery was not unique to this particle and was observed in other ultrasmall Pt nanoparticles (Supporting Information Video S3 and Section S3) and is discussed further in a later section.

### Displacive Deformation of Larger Particles Shows Weakening with Decreasing Size

**Figure 3:**
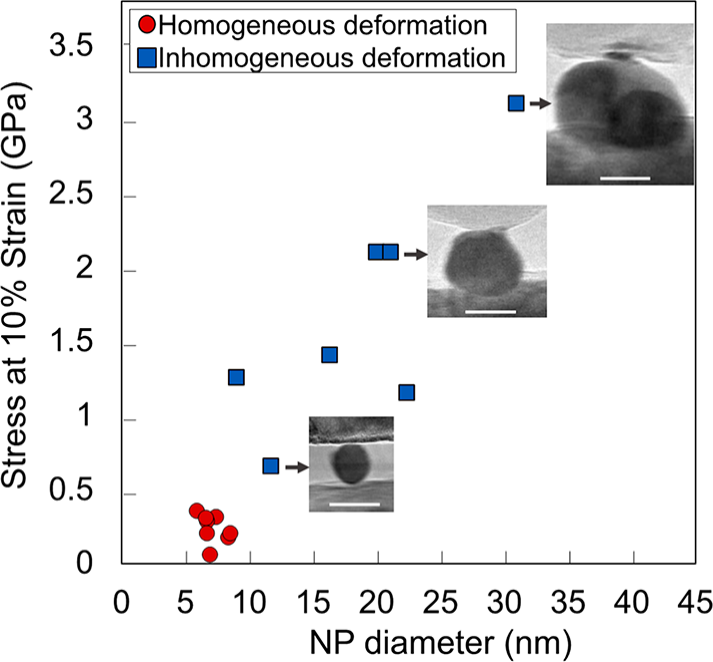
Strength of particles decreases monotonically down to approximately 9 nm, where the deformation changes from inhomogeneous to homogeneous. The stress at 10% strain for all tests is shown as one measure of the strength of the particles. For particles with a clear yield plateau in Figure 1, an average flow stress is shown in Figure S7, but trends are consistent between both measures of nanoparticle strength. The scale bars in the insets are 15 nm.

The strength of larger particles (diameter ≳9 nm) decreased monotonically with decreasing size. A representative value of nanoparticle strength, the stress at 10% strain, is shown in Figure 3. For particles that exhibited a clear yield plateau in Figure 1, a flow stress was computed as the average stress in the plateau and is shown as a function of particle diameter in the Supporting Information Figure S8. The behavior observed in displacive deformation (blue squares in Figure 3) is in contrast to that expected for Coble-creep-like deformation because these larger particles (9–41 nm) clearly demonstrate inhomogeneous deformation with shearing along well-defined slip planes (Supporting Information Videos S1 and S4), rather than the liquid-like deformation associated with the Coble-creep-like model.^26^ The observed behavior is also in contrast to the work of Wang et al.,^30^ which showed Hall-Petch-like strengthening for Pt nanowires that persisted down to 5 nm. This difference in behavior from Wang et al.^30^ is attributed to differences in sample geometry. In the experimental and simulated tensile testing of Wang, the nanowires had straight sides (Figure 2 in ref (30)) and rectangular cross sections (Figure 4 in ref (30)). Therefore, the mean curvature of these wires was significantly lower than typical nanoparticles. Even if they were cylindrical in shape, the Gaussian curvature (a continuum concept) of a sphere is twice as high as for a cylinder due to the two-dimensional curvature. From an atomistic perspective, these nanosized particles have a large number of edge and corner sites to accommodate the large curvature.

There is significant scatter in the strengths of the larger particles (blue points in Figure 3) around the overall trend. This scatter is attributed to two sources: (1) differences in the particles and (2) the stochastic nature of surface-nucleation of dislocations. First, particles were selected for testing to achieve a range of particle diameters, but they clearly differed in other attributes, including crystallographic orientation and particle shape. Crystal orientation modifies the resolved shear stress for defects on particular crystal planes^37^ and also modifies the degree of spatial inhomogeneity of stress.^38^ Particle shape modifies various factors, including stress concentrations at corners,^39^ and the out-of-plane shape of FCC structures can influence which types of defects are favored.^40^ Moreover, the TEM only provides a side-view image of the particle, and therefore, an assumption was made of approximately circular cross-section in the out-of-plane direction. The second factor contributing to the scatter in the measured data is the inherent stochasticity of dislocation nucleation that has been observed in other investigations.^12^ The strength is often represented by using a probability density function^12,41^ reflecting the probabilistic nature of the thermally activated nucleation event.

Despite the scatter in the data, the decreasing trend of strength with decreasing size in the displacive regime reveals the dominant effect of surface curvature on the deformation. The surface nucleation of dislocations has been shown to be sensitive to particle size,^12,14^ crystal orientation,^14^ internal defects,^34^ surface stress,^42^ and surface termination;^43^ however, despite these sensitivities and despite the particle-to-particle variation discussed in the prior paragraph, the effect of size (particle diameter) dominates the behavior. Given the wide range of particle shapes tested, the most consistent effect of decreasing diameter is an increase in surface curvature, suggesting that the curvature of the particle is governing strength in this regime. This is consistent with prior calculations^44^ and simulations^45^ showing the importance of the angle of a corner on dislocation nucleation; sharper angles lead to smaller dislocation line lengths of the embryonic dislocation and thus significantly reduced barriers to nucleation.

Smaller particles and increased surface curvature are also associated with accelerated atomic diffusion because of the Gibbs–Thomson effect on chemical potential, which leads to the depression of the melting point^25,46−49^ and thus higher homologous temperature. The semiempirical thermodynamic model by Kim and Lee^50^ (Supporting Information Section S5) shows that the predicted change in melting temperature is modest. For example, when the nanoparticle diameter shrinks from 30 to 20 nm, the melting temperature changes from 2005 to 1947 K. However, the present investigation could not distinguish whether the reduction in strength was caused by a lowering of the (athermal) barrier to dislocation nucleation or by increased atomic diffusion. Either way, the results show a clear size-dependent weakening as particles shrink from 41 nm to approximately 9 nm, which is suggested to arise due to increased surface curvature that accelerates the surface-nucleation of dislocations.

### Revealing the Driving Force and Atomic-Scale Mechanisms of Homogeneous Deformation in the Smallest Nanoparticles

Particles with sizes below 9 nm (red circles in Figure 3) displayed homogeneous “liquid-like” deformation while maintaining clear crystallinity and exhibited strengths in the range of 143 and 323 MPa. According to the Coble-creep model, a 8.6-nm particle should be approximately 200% stronger than a 6.0-nm particle, yet no size-dependent trend was observed. The strength of all particles was clustered around the same value, with no discernible difference with size.

**Figure 4:**
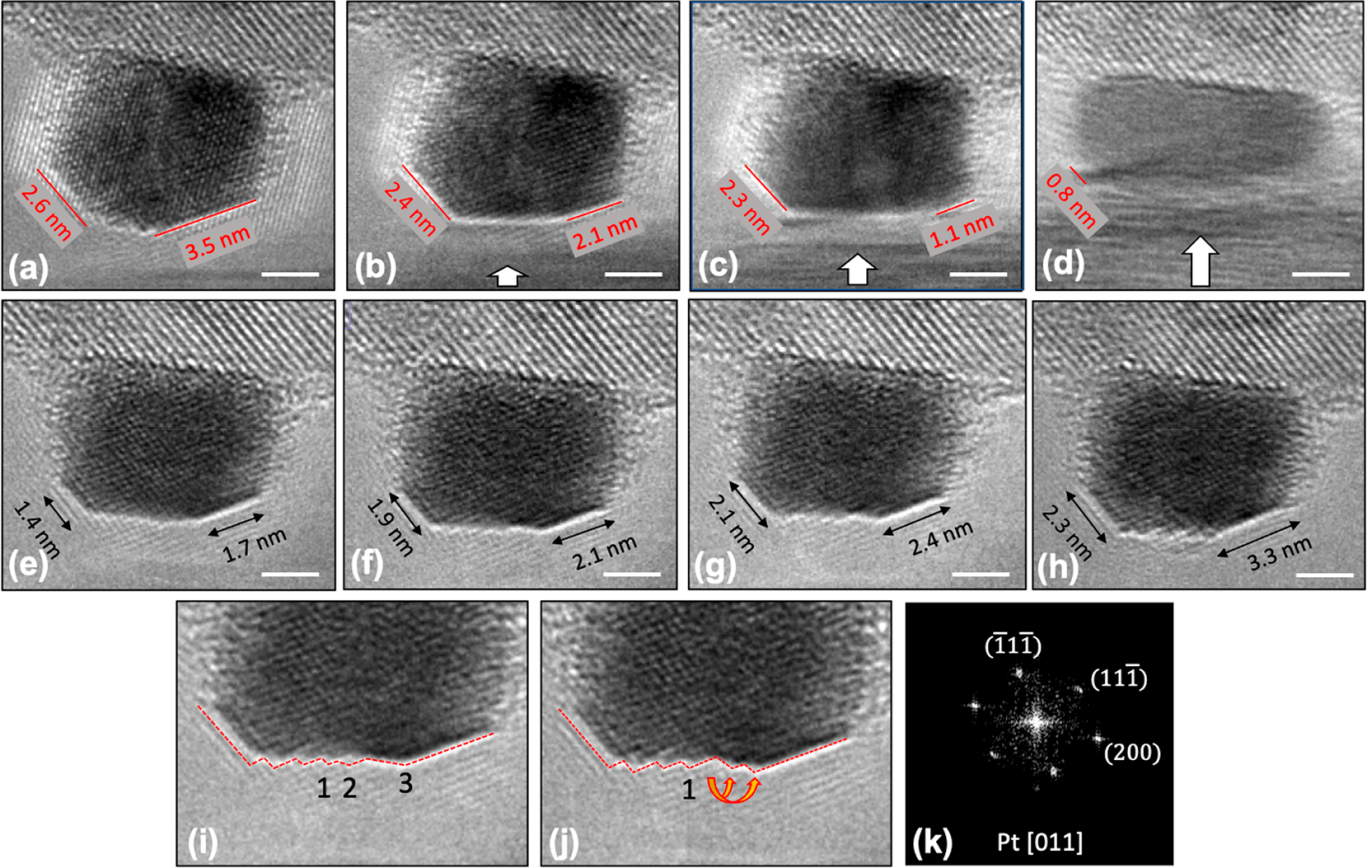
Surface atom migration drives evolution from a stress-free shape to a compressed shape and back. Parts (a–d) demonstrate the shape evolution as the particle is compressed. The initial shape (a) has two {111} facets (red lines in (a)), with a {110}-like facet between them with many surface steps and {100} facets at the sides (between the {111} facets and the substrate). As load is applied (white arrow), the {111} facets shrink in size and material is added to adjacent {100}-type facets on the side walls, which are stress-free (b,c). Panel (d) shows the particle shape at the maximum applied force. After detachment (e–h), atomic hopping causes the growth of the {111} facets and the corresponding shrinking of the high-energy {110}-like facet. The close-up images shown in panels (i) and (j) indicate the surface steps in two consecutive frames of the compression video (0.25 s interval) and illustrate one of many atomic hopping events associated with growth of the low-energy {111} facets. Crystal orientation is given by an fast Fourier transform pattern (k). Scale bars in (a–h) are 2 nm.

To identify the mechanisms of atom migration, a modified test configuration was used (see Methods for details) with an increased spatial resolution. Tests were conducted to strains of 38 and 16% (Figure 4 and Supporting Information Video S4), well beyond the theoretical elastic strain limit in crystalline nanomaterials.^51,52^ During testing, there were no inhomogeneous features or changes in contrast observed that would indicate displacive deformation; however, we acknowledge that the bright-field imaging used in this testing is less sensitive to these changes compared to dark-field. Instead of abrupt changes in particle shape, a significant shape change occurs at the surfaces adjacent to the indenter surface. Then, after removal of the compression, the nanoparticles recover their original shape, including the surface facets, within a few seconds. This shape recovery of small particles was not associated with plastic mechanisms^28,29^ but instead occurs by surface rearrangement, as has been observed in lower-melting FCC metals.^26,31^

Using real-time high-resolution images, the particle’s surface was traced to track the changes in the initial {111} surface facets (red lines in Figure 4a). As the load was applied, the lengths of the {111} surface facets decreased and additional material was added to the {100} facets on the sides of the particle (Figure 4b,c). The particle morphology at the maximum load is shown in Figure 4d corresponding to a 38% strain. After the probe completely detached from the particle, the low-energy {111} surface facets were reconstructed at the expense of other surfaces (Figure 4e), indicating a return to typical surface-energy-driven diffusion from higher-energy facets to lower-energy facets. During the next 20 s after load removal, the {111} corner facets (bottom-left and -right facets) continued to grow from 1.4 and 1.7 nm (Figure 4e) to 2.3 and 3 nm (Figure 4h), respectively. The middle {110}-like facet, with highly unstable atoms, shrank accordingly to minimize the total surface energy of the particle. The TEM used was probe-corrected but not image-corrected; therefore, lattice delocalization and fringe artifacts were present which obscured atom-scale resolution of the edges. However, the growth and change of the interfaces with time is clearly visible when adjacent video frames are compared. A closer look at the middle region (Figure 4i and the corresponding point in Supporting Information Video S4) shows that the surface is composed of atomic steps which dynamically change via a series of atom-hopping events (see Supporting Information Video S4). Three surface steps are specified by numbers in Figure 4i. Step 1 is stable; however, steps 2 and 3 disappear after 0.25 s, and two new steps are created shown by the arrows in Figure 4j. In the new surface configuration, step 2 is recessed, and step 3 is progressed to grow the (111) surface facet. A quantitative analysis of the post-test shape change (Supporting Information Section S6) yields a diffusion constant of 2.8 × 10^–20^ m^2^/s, which is in order-of-magnitude agreement with the value computed by Zhong et al.^53^ This therefore confirms the slow diffusion kinetics of high-melting-temperature platinum, even while demonstrating that large-scale homogeneous deformation is clearly accommodated by surface atom migration.

An intriguing suggestion of these observations is that the deformation behavior is consistent with particle-level energy minimization. Specifically, as the indenter compresses the particle (approximately in displacement control, see Methods), there is a competition between the building up of stored elastic strain energy and the particle’s surface energy. When surface atoms migrate to higher-energy side surfaces, they shrink the particle and thus reduce the strain energy but at the expense of increasing the surface energy. A full consideration of the surface energy would also account for the interfacial energy between the particle and the indenter surface. In the absence of a quantitative calculation, the observations are qualitatively consistent with a process in which the particle takes on a new confined-geometry equilibrium shape. Then, when the compression is removed, the primary driving force goes back to surface energy; the net flux of atoms is from high- to low-energy surfaces, and the particle recovers its original stress-free shape.

## Conclusions

To understand the behavior of surfaces on nanoparticle deformation, we performed in situ compression of platinum with sizes ranging down to the smallest that have ever been tested in a TEM with simultaneous high-resolution measurement of force, as well as particle shape and structure. The results demonstrate three key findings. First, inhomogeneous displacive-like behavior is observed for particles from 41 to 9 nm. This behavior differs from the “smaller-is-stronger” trends seen in platinum nanowires but also differs from the homogeneous deformation that is sometimes associated with “smaller-is-weaker” behavior; instead, the trend is suggested to occur due to the increasing surface curvature, which facilitates dislocation nucleation. Second, below 9 nm, the deformation behavior transitions from inhomogeneous to homogeneous, with measured values of strength between 143 and 323 MPa. No size dependence of strength was observed in this regime, in contrast to the predictions of the Coble-creep-like model; however, the measured 9 nm threshold is considered approximate and requires further investigation. Third, the ultrasmall particles were shown to deform by atomic migration from lower-energy facets to higher-energy facets. The driving force for this migration was suggested as a reduction in strain energy at the expense of surface energy; effectively resulting in a confined-geometry equilibrium shape that deviates from the stress-free equilibrium shape. Taken together, this work sheds light on the mechanisms and driving force by which surfaces govern nanoparticle deformation.

## Methods

**Figure 5:**
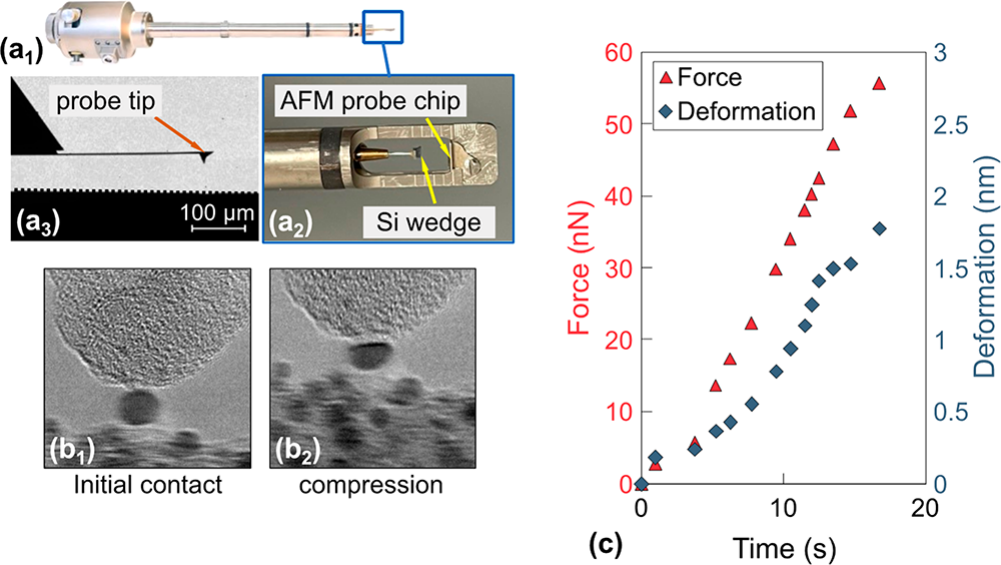
Compression tests were performed using an atomic-force-microscopy probe inside of a transmission electron microscope. An in situ TEM nanomanipulator (a_1_) was mounted with a nanoparticle-decorated Si wedge opposite an AFM probe (a_2_) such that the probe tip could contact the nanoparticles (shown schematically in a_3_). Real-time video is used to extract both the cantilever displacement and contact size (b_1_, b_2_), which is used to compute the force and stress, and the instantaneous particle height, which is used to compute the deformation and strain. Representative data for force and deformation are shown, for the loading phase only, in panel (c).

Bare platinum nanoparticles were synthesized directly on silicon wedges using either wet impregnation (WI) (see ref (35) for details) or physical vapor deposition (PVD) (see Supporting Information Section 7 for details). The nanoparticle-decorated wedges were transferred to a commercial in situ TEM nanomanipulator (biasing manipulator model 1800, Hummingbird Scientific, Lacey, WA) as shown in Figure 5a. The nanomanipulator was modified to include an atomic-force-microscope (AFM) probe as an indenter tip, with a precalibrated^54^ spring constant *k*. Two different types of AFM probes were used: one was composed of silicon with a native oxide coating, and the other was also silicon but with a surface coating of diamond-like carbon. The overall shape of the probe was pyramidal but with a hemispherical apex radius that was larger than the nanoparticles being tested. The compression tests were performed in a TEM (Titan Themis G2 200, Thermo Fisher Scientific, Waltham, MA) at an accelerating voltage of 200 kV. The tests were performed at a constant displacement rate of 0.5 nm/s. The applied force was then computed using Hooke’s law, *F* = *kx*, where *x* is the probe–tip displacement, extracted from post-test video analyses (Figure 5b; further details in Supporting Information Section S8). The true stress was computed as the instantaneous force over the instantaneous contact area, which was computed from a measurement of the contact diameter (*A* = π*D*^2^/4) between the indenter and the particle. Prior work by the authors on similar particles has shown that there can be spatial inhomogeneities in stress depending on shape and orientation,^38^ but the contact stress is considered to be a reasonable characteristic value for representing the stress in the particle.

The real-time video was also used to extract the instantaneous shape and size of the particle (Figures 2 and 4). The deformation of the particle was computed as the difference between the instantaneous height of the particle *h*_i_ and the original height of the particle *h*_0_, while the true strain was computed as ln(*h*_i_/*h*_0_). In this work, analysis was performed on the loading phase of the particles only (Figure 5c). To limit the effect of electron-beam irradiation, the beam intensity was limited to below 10 A/cm^2^, which was sufficiently low to be excluded as a major factor impacting the shape evolution of particles.^26,53^

Two test configurations were used. For most of the tests (Figures 1–3), the AFM probe was mounted on the fixed side, with the nanoparticle-decorated wedge on the piezocontrolled movable side of the nanomanipulator. This enabled force measurements with high accuracy from direct observation of probe–tip deflection. This configuration was used for 16 of the 18 nanoparticles tested, with sizes ranging from 6 to 41 nm. The particle size was defined as the average of the measured width and height of the particle before testing. In order to get higher spatial resolution, two tests were also performed (Figure 4), with the positions of the probe and nanoparticle-decorated wedge reversed. Here, the nanoparticles were mounted on the nonmovable portion of the apparatus, thus reducing vibration of the nanoparticles. The force was not measured in this modified test configuration, but this latter setup was ideal for tracking the surface atom rearrangements during and after the indentation.

## Supporting Information Available

Inhomogeneous deformation of a 20.0 nm Pt NP (video speed 2×) (MP4)
Homogeneous deformation of a 8.6 nm Pt NP (video speed 2×) (MP4)
Homogeneous deformation of a 7.5 nm Pt NP (MP4)
High-resolution compression test of a Pt NP with surface atomic transportation during and after compression (MP4)
Liquid-like behavior of a small nanoparticle and high to low curvature evolution (MP4)
In addition to the above videos, the Supporting Information PDF contains the following sub-sections: representative images showing the angle of nanoparticle shearing (section S2); homogeneous deformation and shape recovery of a 7.5 nm nanoparticle after a series of separate consecutive compressions tests (section S3); average flow stress of Pt nanoparticles (section S4); theoretical calculation of melting temperature vs size in Pt nanoparticles and nanowires (section S5); calculation of diffusion rate in a small Pt nanoparticle (section S6); description of the process of physical vapor deposition of Pt nanoparticles on a silicon substrate (section S7); formulation to calculate stress and strain from the TEM frames (section S8) (PDF)
