# Supplementary material for: Size-Dependent Role of Surfaces in the Deformation of Platinum Nanoparticles
Source: ACS Nano. 2023 Apr 26;17(9):8133–40. doi: 10.1021/acsnano.2c11457 (PMC10173686; doi:10.1021/acsnano.2c11457)
Supplement: Supplementary file 6 — nn2c11457_si_006.pdf [file nn2c11457_si_006.pdf]

Size-dependent role of surfaces in the deformation of platinum nanoparticles - Supporting Information

Soodabeh Azadehranjbar,† Ruikang Ding,† Ingrid M. Padilla Espinosa,‡ Ashlie Martini,‡ and Tevis D.B. Jacobs*,†

†*Department of Mechanical Engineering and Materials Science, University of Pittsburgh, Pittsburgh, PA 15261, USA*

‡*Department of Mechanical Engineering, University of California, Merced, Merced, CA 95340, USA*

E-mail: tjacobs@pitt.edu

Phone: +1 412 624 9736

Section S1: Supporting videos S1 - S5

Still frames of the supporting videos showing the size dependent deformation mechanisms and the role of surface in representative nanoparticles.

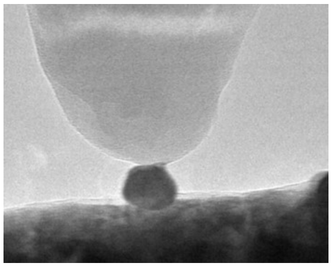

Figure S1: Still frame of Video S1 showing inhomogeneous deformation of a 20.0-nm Pt NP (video speed 2x).

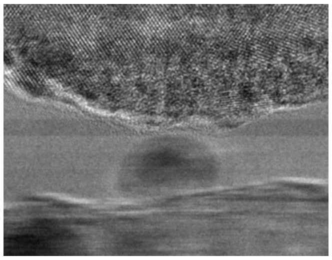

Figure S2: Still frame of Video S2 showing homogeneous deformation of a 8.6-nm Pt NP (video speed 2x).

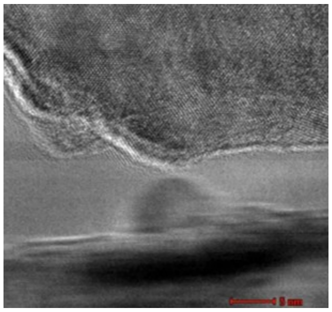

Figure S3: Still frame of Video S3 showing repeated homogeneous deformation of a 7.5-nm Pt nanoparticle.

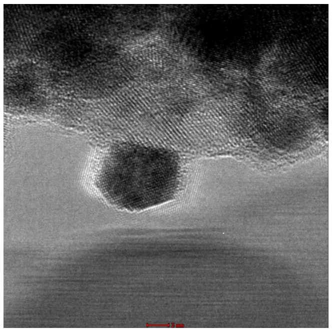

Figure S4: Still frame of Video S4 showing a high resolution compression test of a Pt nanoparticle with surface atomic transportation during and after compression

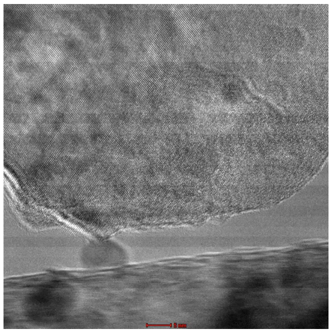

Figure S5: Still frames of Video S5 showing liquid-like behavior of a small nanoparticle, and high to low curvature evolution.

Section S2: Representative images showing the angle of shearing

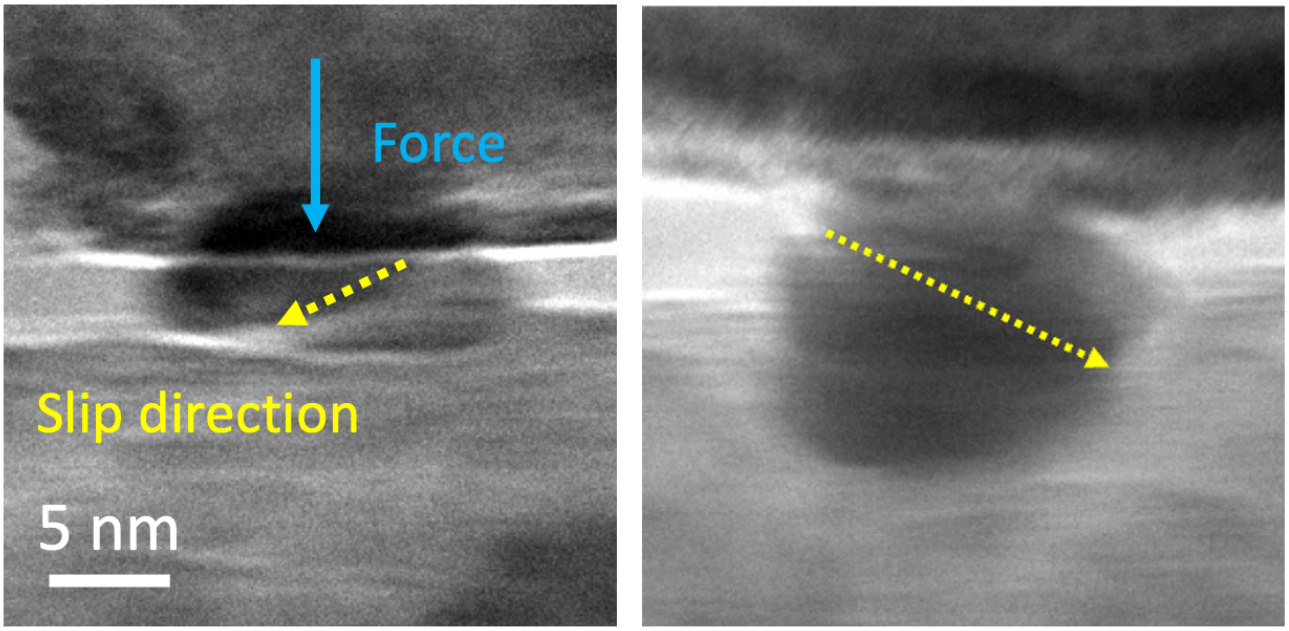

Figure S6: Representative images showing the force and the angle of shearing.

Section S3: Homogeneous deformation and shape recovery of a representative nanoparticle below 9 nm

One of the nanoparticles (identified as 7.5 nm in the main text, Fig. 1) was compressed many times in order to understand the repeatability of shape recovery. While only one test was quantitatively analyzed for stress and strain, this figure shows a progressive array of still images representing multiple compressions. Seven separate compression tests were performed in the same video, and for each test there is a frame before testing, at maximum compression, and after testing. This set of figures corresponds to supporting Video S3.

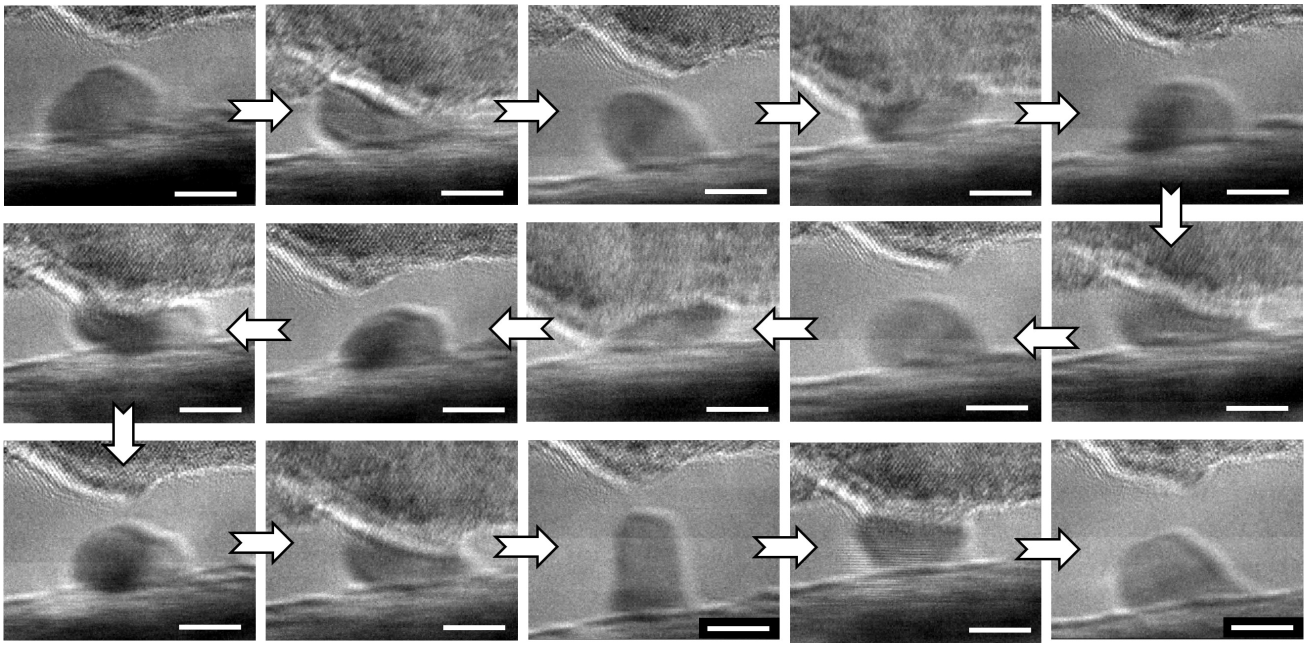

Figure S7: Repeatable homogeneous deformation of a 7.5 nm Pt nanoparticle. Scale bars are 5 nm.

Section S4: Flow stress

Some particles exhibited a clear yield plateau (main text, Fig. 1) and therefore allowed the calculation of the average stress during yielding/ While other tests had to be stopped prior to yielding due to limitations of the experimental setup. Consequently, while the stress at 10% strain (main text, Fig. 3) can be computed for all tests, a flow stress cannot be. For those tests of particles greater than 9 nm that DID show a yield plateau, Fig. S8 shows their flow stress. The flow stress was computed as the average stress in the plateau and it is shown as a function of particle diameter. The trends are consistent with those of the stress at 10% strain

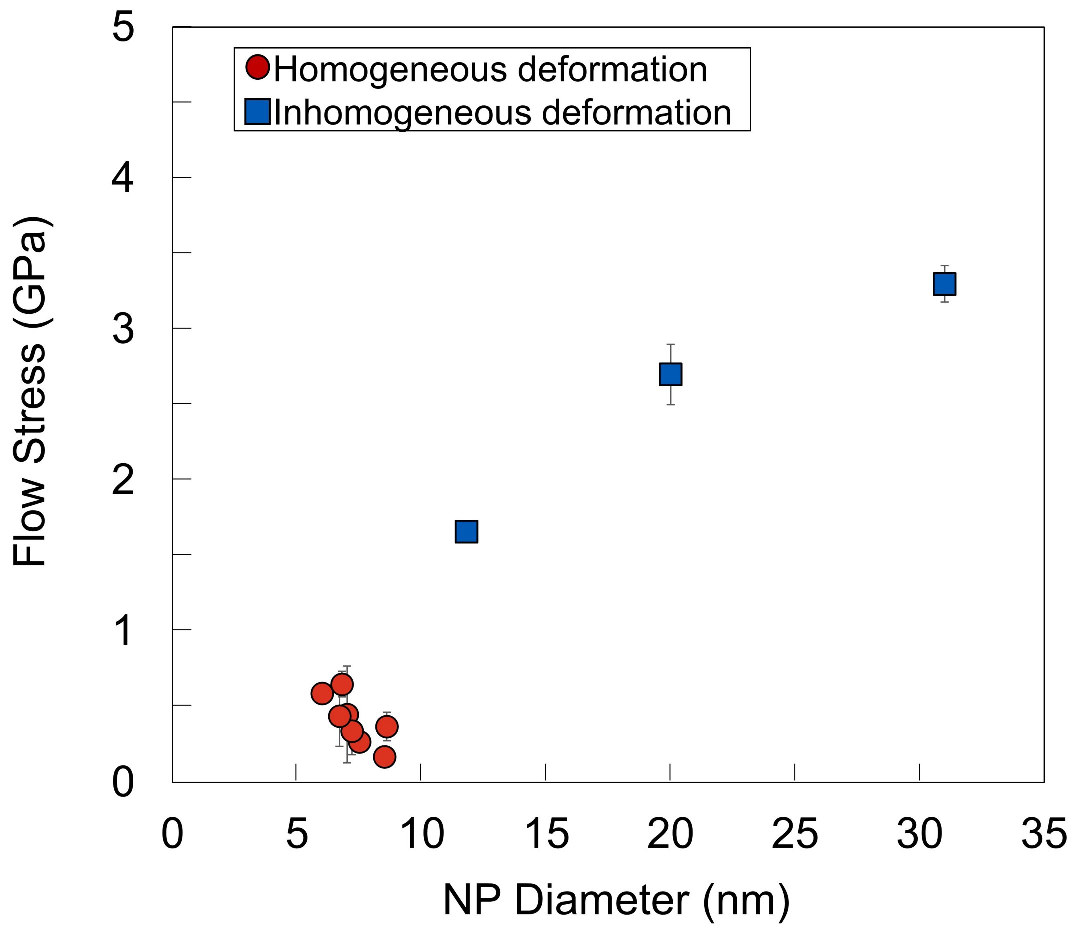

Figure S8: Average flow stress for particles with a clear yield plateau. The strength of particles decreases monotonically down to a critical size of 9 nm, where the deformation changes from inhomogeneous to homogeneous.

Section S5: Melting temperature vs size in nanoparticles and nanowires

The semi-empirical thermodynamic model by Kim and Lee[1] was used to estimate the predicted variation in melting temperature ΔT_m with size in Pt nanoparticles and Pt nanowires, shown in equations 1 and 2 respectively.

$$\Delta T_m = \frac{2}{r}\frac{V_s T_m}{\Delta H_m}\Delta\gamma\left(1+\frac{r_e}{r}\right)^2 \tag{1}$$

$$\Delta T_m = \frac{1}{r}\frac{V_s T_m}{\Delta H_m}\Delta\gamma\left(1+\frac{r_e}{r}\right) \tag{2}$$

where γ is the surface energy, V_s is the room temperature solid molar volume, ΔH_m is the latent heat of melting, T_m is the melting point of the bulk material, r_e is the first nearest neighbor distance for crystalline materials, and r is the particle radius or the cylinder radius in nanowires.

The parameters for Pt are given in Table S1.

γ	$0.337\ \frac{J}{m^2}$
V_s	$9.09 \times 10^{-6}\ \frac{m^3}{mol}$
ΔH_m	$22.18\ \frac{kJ}{mol}$ *
r_e	0.277 nm

Table S1: Physical properties of Pt to predict the melting temperature related to size for nanoparticles and nanowires.[1]

The predicted melting point depression of Pt nanoparticles and nanowires is shown in Fig. S9

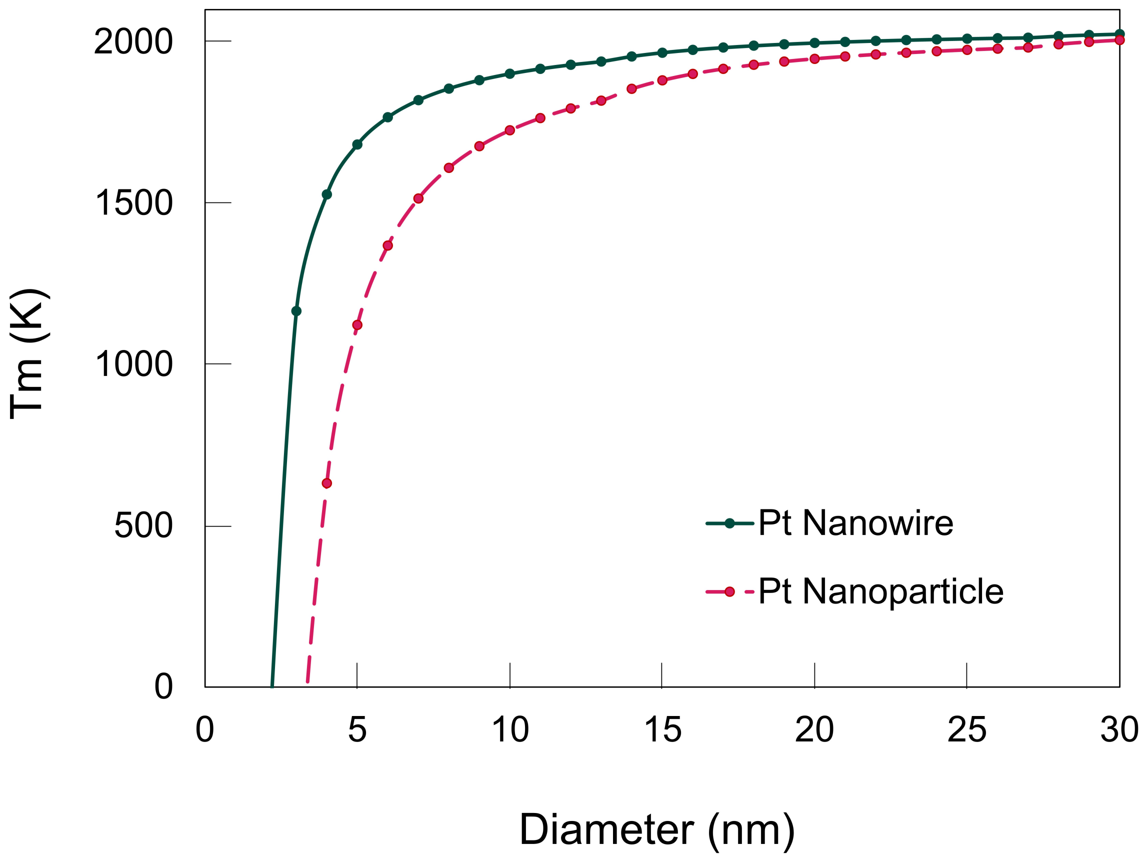

Figure S9: Predicted melting temperature of Pt nanoparticles shown as the dashed dark pink line and Pt nanowires shown as a continuous green line.

Section S6: Calculation of diffusion rate in a small Pt nanoparticle

According to the classical curvature-driven shape equilibration theory[2], atoms on high-curvature surfaces possess higher chemical potentials and tend to move toward the lower-curvature surfaces with lower chemical potentials. The diffusion rate or diffusion coefficient (D_s) of atoms in the surface is calculated according to equation 3[2,3].

$$D_s \sim \frac{R^3 h k_B T}{\tau \gamma \omega \delta_s} \tag{3}$$

Where R is the radius of curvature, h is recession height, τ is the relaxation time, δ_s is the surface layer thickness, γ is the surface energy, and ω is the atomic volume.

To understand the driving force for atom migration, we first compute the diffusion coefficient of Pt from our experiments. The curvature evolution of a small nanoparticle is shown in Video S5. Still frames of the curvature for this nanoparticle are shown in Fig. 3.

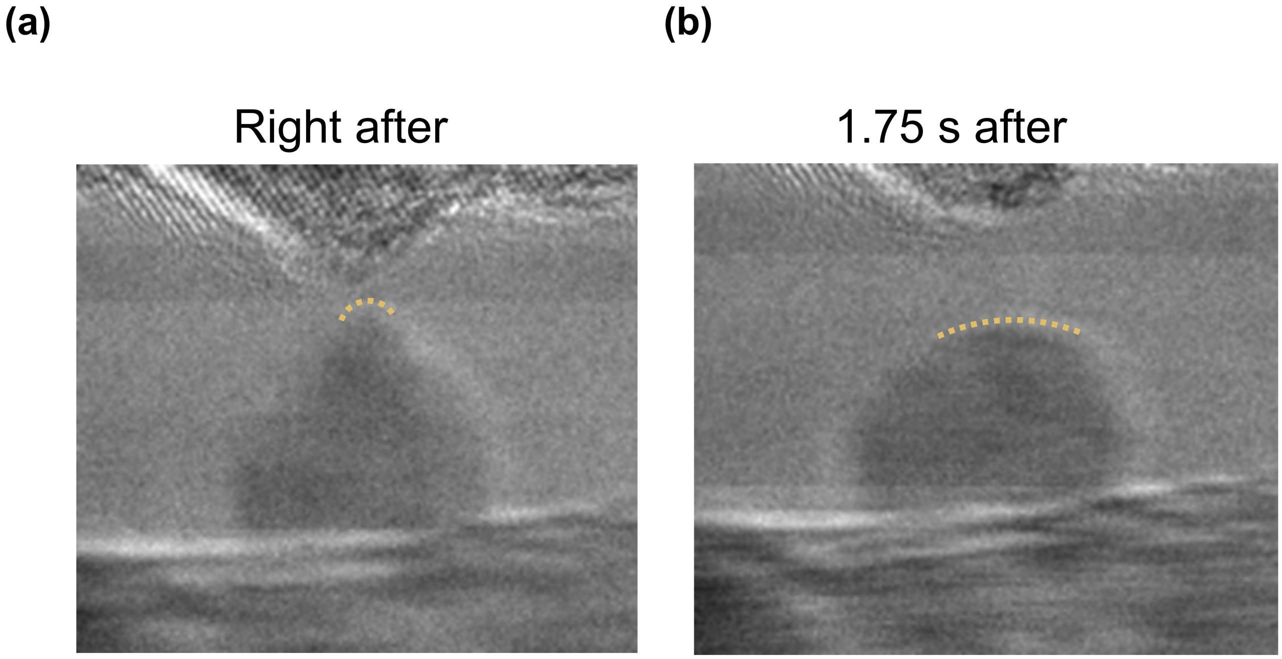

Figure S10: Curvature evolution in a small Pt nanoparticle after probe deattachment

The Pt nanostructure had a sharp local radius at the point of detachment from the indenter (radius R=1.1 nm). Then, there was continuous change of tip morphology, where the recession rate started fast (2.3 nm/s) and rapidly diminished to 0.66 nm/s within only 1.5 s, while the radius of curvature approached 5 nm. These values were used to calculate the diffusion rate as D_s = 2.8 $\times 10^{-20}$ $\frac{m^2}{s}$. This value was confirmed by applying the measured D_s and Mullins equation to the shape-relaxation (shape equilibration) of another nanoparticle (shown in Fig. 2f (1.2 nm) to Fig.2k (2.1 nm)). The observed relaxation time (7.75 s) for such shape evolution is well within the range calculated based on the Mullins equation (2-18 s). The measured value of D_s = 2.8 $\times 10^{-20}$ $\frac{m^2}{s}$ is in reasonable agreement with values for Pt nanotips previously reported, D_s = 1.8 $\times 10^{-20}$ $\frac{m^2}{s}$[4]. The above analysis confirms the sluggish slow diffusion kinetics of high-melting-temperature Pt, even though surface atom migration was clearly visible in the mechanical testing.

Section S7: Physical Vapor Deposition of Pt nanoparticles

The $\sim 1 - \mathrm{mm}^3$ wedge-like silicon substrates (< 200 nm Plateau, Bruker, Billerica, MA) were cleaned by oxygen plasma before any treatment. The top surface of the wedges was then coated by a 20-nm CeO_2 layer by sputtering (Nexdep, Angstrom Engineering, Kitchener, Canada). This coating is necessary as CeO_2 layer is more thermally stable than silicon. Then, platinum layers of 0.5 nm and 1 nm thickness were deposited at a rate of 0.03 nm/s on the coated wedges by electron beam evaporation (MEB550S, Plassys, Marolles–en–Hurepoix, France). Finally, the samples were annealed at 630 °C and 800 °C for 30 min in air atmosphere (Thermolyne 1200 °C 7 in×5 in×10 in, Thermo Scientific, Waltham, MA). The heating rate for all samples were controlled to be about 10 °C/min. Annealing time was controlled in the level of 30 min to avoid overheating. The annealing time is just a minor variable in nanoparticle size control as nanoparticles rapidly evolve to a relatively stable state at high temperature, unless heating time is too long, e.g., > 3 hr and nanoparticles are over-coarsened[5]. Different synthesis recipes provide nanoparticles at a wide range of sizes spanning from 5 nm to > 50 nm (low annealing temperature for small nanoparticles, and high annealing temperature for large nanoparticles), which covers the size range studied in this research.

Section S8: Calculation of stress and strain

The applied load F is calculated using the Hooke's law by measuring the AFM tip displacement Δx multiplied by the spring constant k of the tip (Fig. S11a-b).

$$F = k\Delta x \tag{4}$$

To study the strength of the particles, we calculated the true stress σ by dividing the applied load F by the contact area of the particle and the tip. A circular contact region was presumed, and its radius r was measured at each frame (Fig. S11d).

$$\sigma = \frac{F}{\pi r^2} \tag{5}$$

The true strain ϵ was calculated by measuring the initial height h_0 and instant height h of the particle as shown in panels Fig. S11c-d.

$$\epsilon = ln\frac{h}{h_0} \tag{6}$$

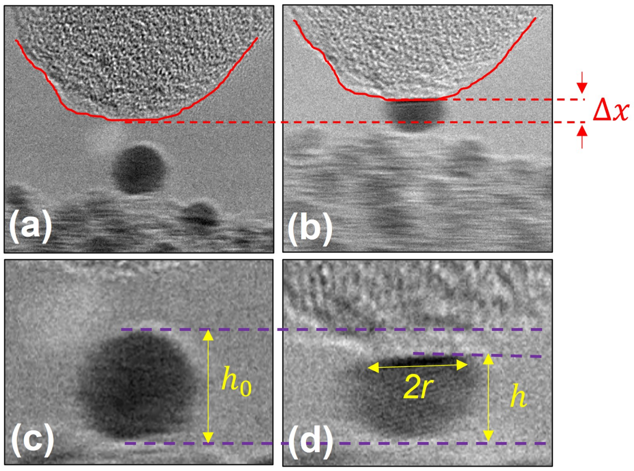

Figure S11: The reference point is the pre-contact position of the AFM probe, which is where it sits when it is under zero load. All deflections from that point represent a change in position that indicates a load is applied.

References

(1) Kim, E.-H.; Lee, B.-J. Size dependency of melting point of crystalline nano particles and nano wires: A thermodynamic modeling. *Met. Mater. Int.* **2009**, *15*, 531–537.

(2) Mullins, W. W. Mass transport at interfaces in single component systems. *Metall. Mater. Trans. A* **1995**, *26*, 1917–1929.

(3) Tian, L.; Li, J.; Sun, J.; Ma, E.; Shan, Z.-W. Visualizing size-dependent deformation mechanism transition in Sn. *Sci. Rep.* **2013**, *3*, 2113.

(4) Zhong, L.; Sansoz, F.; He, Y.; Wang, C.; Zhang, Z.; Mao, S. X. Slip-activated surface creep with room-temperature super-elongation in metallic nanocrystals. *Nat. Mater.* **2017**, *16*, 439–445.

(5) Zhou, L.; Poggesi, S.; Casari Bariani, G.; Mittapalli, R.; Adam, P.-M.; Manzano, M.; Ionescu, R. E. Robust SERS Platforms Based on Annealed Gold Nanostructures Formed on Ultrafine Glass Substrates for Various (Bio)Applications. *Biosensors* **2019**, *9*, 53.
